# Tetraploid–diploid mosaicism in a patient with pigmentary anomalies of hair and skin: a new dermatologic feature

**DOI:** 10.1002/ccr3.1114

**Published:** 2017-11-29

**Authors:** John Paul Schacht, Elisha Farnworth, Jacob Hogue, Luis Rohena

**Affiliations:** ^1^ Department of Pediatrics Division of Clinical Genetics Columbia University Medical Center New York City New York; ^2^ Flight Medicine Clinic 92nd Medical Group Fairchild Air Force Base Tacoma Washington; ^3^ Department of Pediatrics Division of Medical Genetics Madigan Army Medical Center Fort Lewis Washington; ^4^ Department of Pediatrics Division of Medical Genetics San Antonio Military Medical Center San Antonio Texas; ^5^ Department of Pediatrics Division of Medical Genetics University of Texas Health Science Center at San Antonio San Antonio Texas

**Keywords:** Diploid/tetraploid, hyperpigmentation, hypopigmentation, mosaicism, pigmentary dysplasia

## Abstract

Tetraploid–diploid mosaicism in humans is exceedingly rare. We present an 11‐year‐old boy with tetraploid–diploid mosaicism and coexistent hair hypopigmentation with skin hypo‐ and hyperpigmentation. This case expands the current literature as we are not aware of previous documentation of this unique combination of pigmentary anomalies.

## Graphical abstract

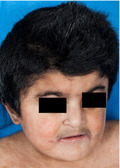

## Introduction

Complete tetraploidy can be defined as four chromosome sets in one cell and usually occurs secondary to nondisjunction during the first mitosis in a zygote 1. It has been reported in spontaneous abortions, but is rare in live‐born infants 2. Affected patients have severe congenital anomalies and a limited life expectancy 2. Most surviving patients are tetraploid–diploid mosaics. Such patients are an example of mixoploidy, a condition characterized by the coexistence of normal diploid cells with cells containing a multiple of the normal haploid chromosome number 3. Tetraploid–diploid mosaicism in living patients has been reported in at least 28 cases since 1967 2, 3, 4, 5, 6, 7, 8, 9, 10, 11, 12, 13, 14, 15, 16, 17, 18, 19, 20, 21, 22. Common clinical features described in the literature include low birthweight, hypotonia, craniofacial abnormalities, and intellectual disability. Dermatologic manifestations of this genetic abnormality have only been reported in seven cases 2, 3, 8, 10, 21, 23.

We present a patient with tetraploid–diploid mosaicism: an 11‐year‐old boy who has coexistent hypo‐ and hyperpigmentation of his skin and hypopigmented gray streaks throughout his hair. This combination has not been described previously in tetraploid–diploid mosaicism.

## Clinical Report

Our patient is an 11‐year‐old boy who has profound intellectual disability, severe developmental delays, and multiple congenital anomalies. This infant male was born at 33 weeks’ gestation via cesarean section to a gravida 5 now para 4, 33‐year‐old mother and 39‐year‐old father with no consanguinity. Maternal ethnicity is unknown, and paternal ethnic background is of Mexican ancestry. Both parents were in good health, and mother was without medical problems and denied medication use or known exposures during pregnancy. This is the first child of this union; however, the patient has three maternal half‐siblings who are of good health and without dysmorphic features.

The pregnancy was complicated by IUGR and oligohydramnios discovered at 30 weeks. The delivery was uncomplicated, and the patient did not require respiratory support but was admitted to the NICU due to gestational age and very low birthweight (1280 g). Birthweight, occipital–frontal circumference and length were less than the 3rd centile. An initial examination was notable for adducted thumbs with clinodactyly of the fifth digits, bilateral cryptorchidism, and facial dysmorphism, spurring an evaluation for Cornelia de Lange syndrome. NIPBL and SMC1A gene sequencing were normal. Initial chromosome analysis from peripheral blood lymphocytes demonstrated a normal male complement, 46,XY. Newborn screens numbers 1 and 2 were normal. FSH and LH levels were normal at 1 week of age. Facioscapulohumeral dystrophy deletion mutation testing was negative. He passed his newborn hearing screen. An echocardiogram showed normal findings except for PFO and benign pulmonary branch stenosis. Initial ophthalmologic evaluation was normal. Renal US showed three cortical renal cysts bilaterally and mild left‐sided pelviectasis. VCUG showed Grade 3 vesicoureteral reflux into a partially duplicated collecting system with a partial ectopic ureter on the right and a dilated posterior urethra. He had normal renal function. Head ultrasound showed choroid plexus cysts**.** He remained in the NICU for roughly 1 month due to difficulty feeding and difficulty gaining weight. He was discharged on amoxicillin for urinary tract infection prophylaxis.

**Figure 1:**
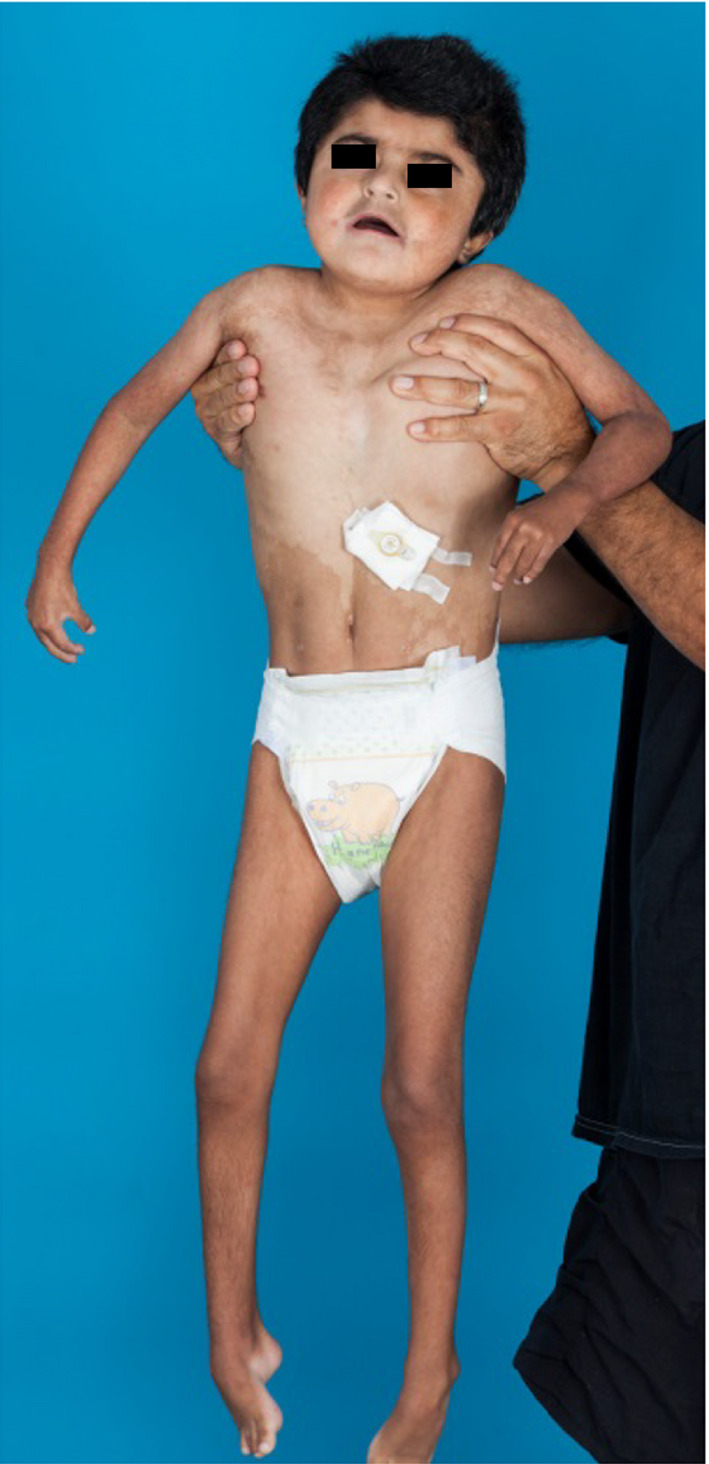
Coexistent hyper‐ and hypopigmentation, most prominent on the abdomen and trunk.

**Figure 2:**
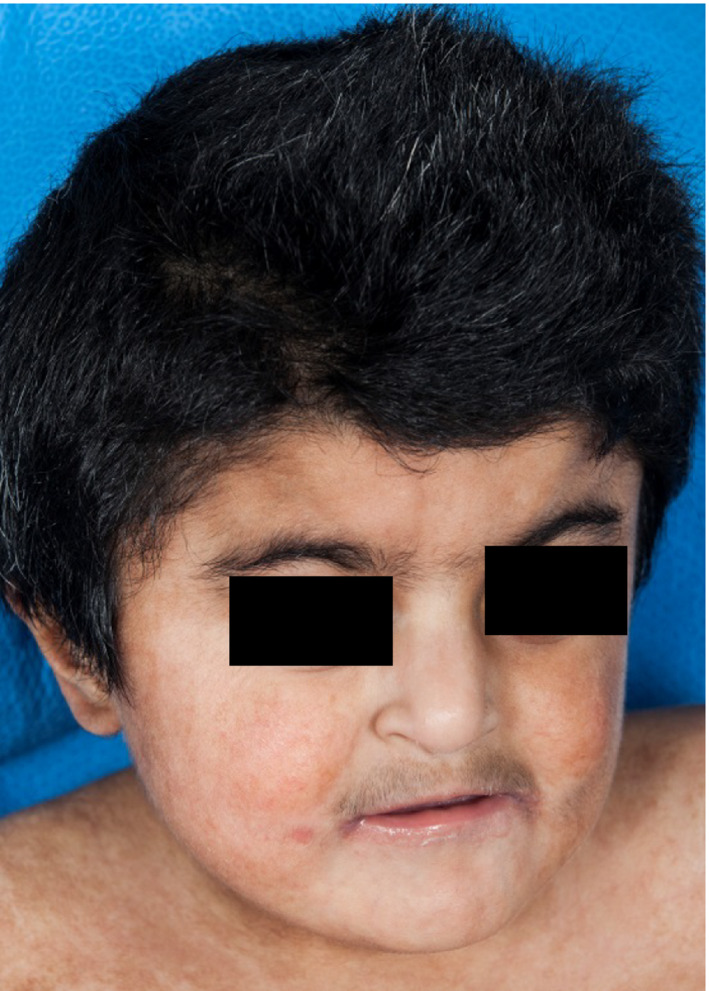
Hair and skin pigmentary dysplasia, along with hypertrichosis and synophrys. (All photographs show patient at age 7).

**Figure 3:**
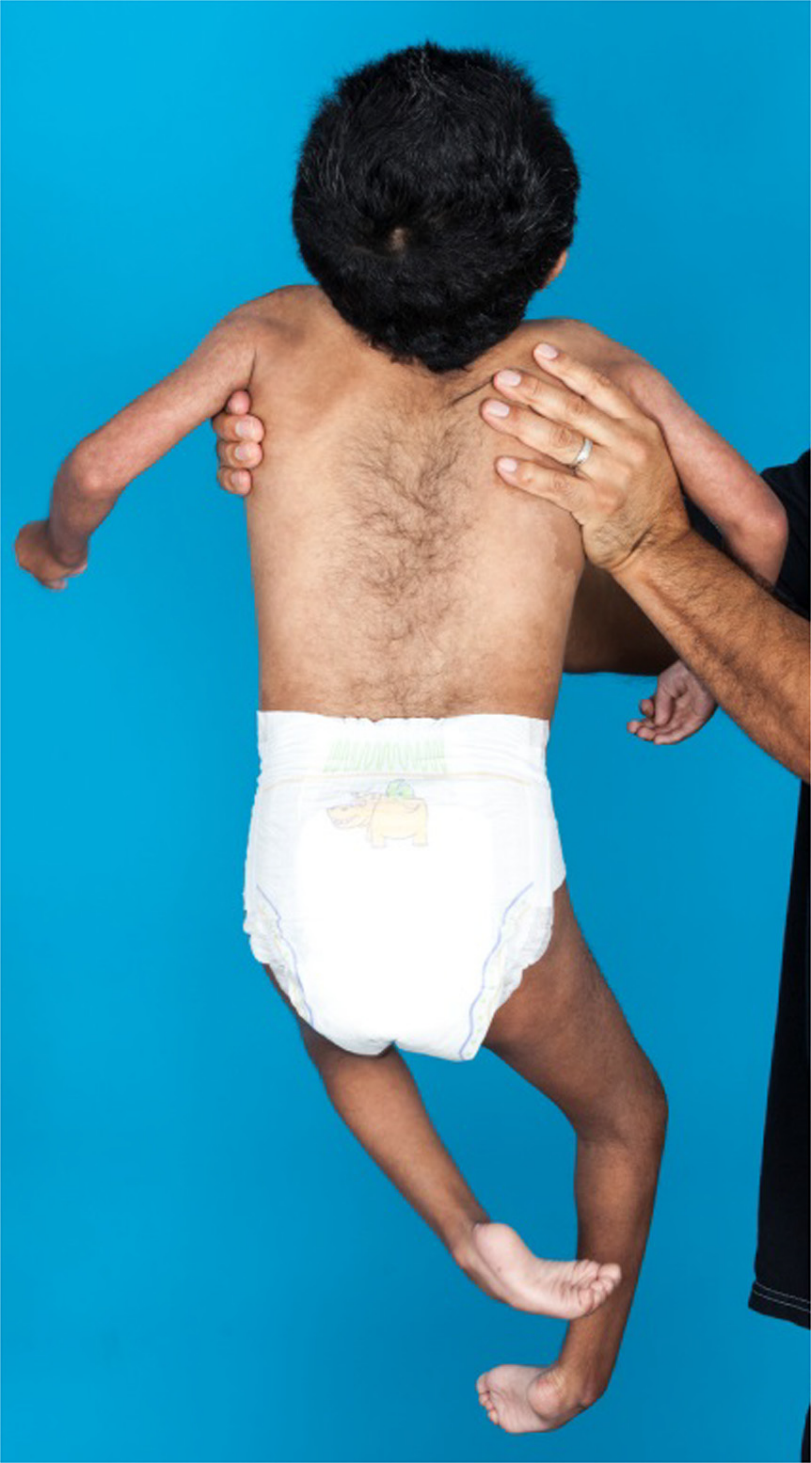
Prominent hypertrichosis covering patient's back.

**Figure 4:**
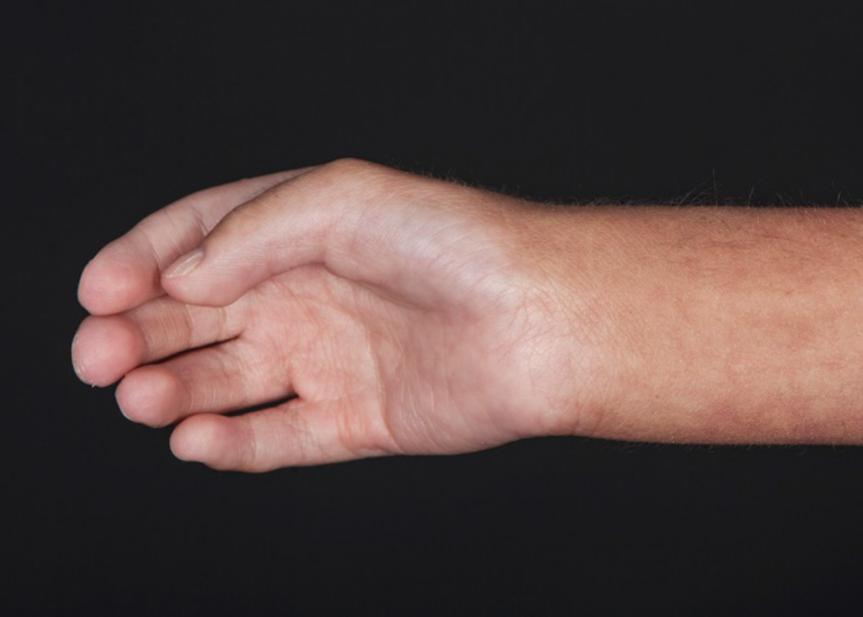
Right thumb contracture.

**Figure 5:**
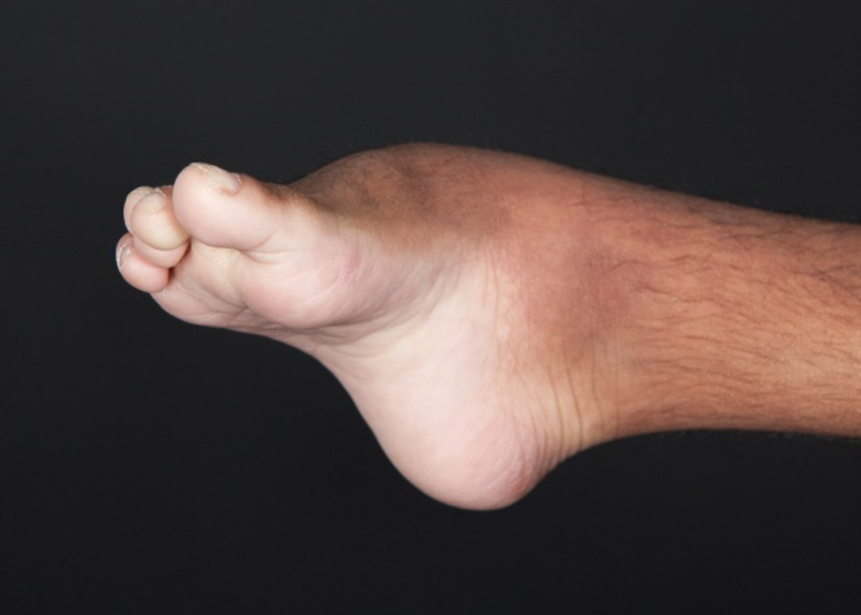
Foot deformity.

Following NICU discharge, the patient continued to have poor growth and global developmental delay. Height, weight, and OFC were well below the 1st centile despite increased caloric intake. He was also noted to have continued bilateral cryptorchidism. A skin tag above his gluteal crease was discovered prompting spinal US which showed findings concerning for a tethered cord. He was noted to have mild asymmetry with a larger diameter of his left leg and a slightly longer left arm. He had left‐sided torticollis with a left‐sided sternocleidomastoid nodule. He also kept his fists clenched with his fingers wrapped around his thumbs. He would grab at objects with his fingers, but would keep his thumbs against his palms, requiring corrective braces. He was referred to physical therapy and neurology (Figs 1, 2, 3, 4, 5).

At age of 4 months, neurology noted microbrachycephaly, hypertonic lower extremities, and bilateral thumb contractures concerning for a central etiology. They also noted dysmorphic facial features including low set ears, flattened and elongated philtrum, downturned corners of the mouth, and a flat nasal bridge. He had midline hair whorls and hirsutism of his bilateral arms. He was also noted to have an abnormally low posterior hairline and bilateral inverted nipples. Cervical spine series showed no vertebral abnormalities. MRI of the lumbar spine to evaluate for tethered cord showed the conus medullaris terminating abnormally low at the superior endplate of L4, without any dermal sinus. This finding was thought to represent a tethered cord versus low conus, and he was followed clinically. He is being evaluated by neurosurgery at the time of this writing. An accessory spleen just anterior to the spleen was incidentally identified, and it was discovered that the patient actually had a horseshoe kidney.

His torticollis continued despite physical therapy and he was noted to have significant right‐sided positional plagiocephaly. Computed tomography of his head to rule out craniosynostosis at age of 7 months showed severe plagiocephaly with a right‐sided positional component that was inadequate to explain the degree of plagiocephaly. Multiple wormian bones were visualized. All sutures appeared patent but the bilateral coronal sutures appeared relatively uniformly smooth and narrow compared with the sagittal and lambdoid sutures. Other notable findings included possible partial absence of the splenium of the corpus callosum, slightly enlarged ventricular system, especially the 3rd ventricle, temporal tips, and lateral ventricles. He required a corrective helmet for his plagiocephaly. He was noted to have bilateral passively correctable foot deformities consisting of neutral hindfeet, supinated forefoot, valgus midfoot, and flexion of the 4th and 5th rays attributed to muscular spasticity. He required bilateral ankle foot orthoses. Pelvic imaging showed coxa valga of both hips. As he aged, he developed a thoracolumbar dextroscoliosis.

Bilateral blue‐gray sclera was noted at 6‐month visit, a condition that can be seen in several syndromes but can also be a normal variant. Chest X‐ray to rule out osteogenesis imperfecta (OI) showed 13 rib pairs, partial posterior fusion of the right 10th and 11th ribs, and multiple mildly osteopenic ribs bilaterally. The cardiothymic silhouette and lung fields were normal. Skeletal survey to rule out OI showed bilaterally shallow acetabuli with abnormally small acetabular angles, a nonspecific finding, and no evidence of fractures.

Repeat hearing test via optoacoustic emissions was normal at 7 months. Sedated ABR at 12 months showed mild bilateral sensorineural hearing loss. Repeat sedated ABR with toneburst at 25 months showed severe bilateral sensorineural hearing loss. He was evaluated by ENT for recurrent episodes of acute otitis media and noted to have stenotic bilateral external auditory canals and required bilateral pressure equalizing tubes to be placed multiple times.

At 9 months of age, emesis with feeds prompted an upper GI series to rule out pyloric stenosis, tracheoesophageal fistulas, and other anatomic abnormalities; the study showed gastroesophageal reflux, but no anatomic abnormalities. Nuclear medicine imaging showed moderate‐to‐severe gastric emptying delay. Videofluoroscopic speech swallow study for recurrent nasal reflux demonstrated oropharyngoesophageal dysphagia with aspiration, and a feeding regimen for the patient was created per speech therapy recommendations. The patient also had significant constipation possibly secondary to a small anus. His reflux and severe atopic dermatitis were concerning for milk protein intolerance, and he was switched to hypoallergenic formula. He eventually required gastrostomy tube secondary to multiple aspirations leading to pneumonia. He continued to have a markedly low weight despite adequate caloric intake.

At age of 15 months, he was referred to pediatric endocrinology due to concerns for poor sexual development, notably a flattened scrotum and thin phallus. Endocrinology workup showed normal TSH, free T4, and IGF‐1. His growth velocity was also normal at age of 25 months at 10 cm/year.

As an infant, skin examination showed diffuse hyperpigmented macules of varying sizes, hyperpigmented patches with swirl patterns on his bilateral flanks, hypo‐ and hyperpigmented whorls, and patches of hypo‐ and hyperpigmentation on all four extremities. He was also noted to have hypopigmented swaths of skin on his torso and arms that subsequently evolved into streaky brown hyperpigmentation. He was noted to have multiple gray scalp hairs, thick eyebrows, and hypertrichosis on his back and arms. At 2 years, a repeat chromosome analysis with a skin biopsy sample revealed 8% of cells with a tetraploid configuration 92,XXYY/46,XY. In 3/50 cells, there were two missing chromosomes 15 and 20, and one cell had an extra chromosome 7.

Other features that became prominent as he grew older included a short neck, pectus excavatum, right‐sided polythelia, bilateral microtia, synophrys, mild soft tissue syndactyly of fingers 3–4, broad finger tips, and short middle palmar creases. His testicles failed to descend, and he required bilateral orchiopexy.

At age of 6 years, he had staring episodes and abnormal head and hand movements. An EEG showed generalized spike and polyspike slow wave complexes as well as focal spike and polyspike slow waves, placing him at risk for focal and secondarily generalized seizures. Background activity was also excessively low for age suggesting encephalopathy. His hamstrings and gastrocnemius muscles were hypertonic but his other peripheral and central muscle groups were hypotonic. He had diffuse hyperreflexia. MRI of the brain showed focal cortical dysplasia of the left inferior temporal gyrus, significant microcephaly with prominence of the 3rd greater than 4th ventricles, a small corpus callosum with underdeveloped body and splenium, mild funnelling of the aqueduct of Sylvius, and a developmental venous anomaly of the right posterior occipital lobe. The cerebellar vermis was hypoplastic and the enlarged 4th ventricle communicated with a moderate‐sized infra‐ and retrocerebellar CSF space or cyst, findings consistent with Dandy Walker malformation. MRI of the cervical spine to evaluate for bony abnormalities due to torticollis showed narrowing of the cervical spinal cord, acute dextrocurvature, and at the craniocervical junction, craniocervical stenosis. The dens created a mass effect on the cervicomedullary junction and caused flattening and deformity of the ventral cord near the cervicomedullary junction. The articulation between C1 and C2 on the left was abnormal with a more vertical than horizontal orientation. C2 and C3 had partial fusion of posterior left elements. The thoracic and lumbar spinal cord were relatively narrow compared with the cervical cord. Dextrocurvature of the thoracic spine was noted. The horseshoe kidney was incidentally noted to have increased in size with an increase in the number of cysts and featured a complex cyst. A renal and bladder ultrasound demonstrated that the cysts had actually decreased in size, while the testicles were atrophied bilaterally.

At 11 years old, the patient has profound intellectual disability with global developmental delays, epilepsy, moderate‐to‐severe sensorineural hearing loss, spastic diplegic cerebral palsy, and persistent poor growth requiring gastrostomy tube feedings. Other medical issues include severe obstructive sleep apnea, lagophthalmos, high myopia, constipation, chronic bronchitis, recurrent pneumonias, and persistent asthma. He was briefly hypertensive early in life and required pharmacologic therapy, but is now normotensive. He makes nonspecific mama and dada sounds. He is able to roll and sit‐up on his knees unassisted.

The content of this manuscript is not considered research at our institution and instead falls in the realm of routine clinical care.

## Discussion

The phenotype of tetraploid–diploid mosaicism is variable and often consists of developmental delays, intellectual disability, and growth retardation. Other documented features include hypotonia, skin pigmentary dysplasia, seizures, and body asymmetry. Our patient had a similar presentation but in addition had pigmentary dysplasia of his hair.

Pigmentary skin changes have been associated with chromosomal mosaicism 24. At least two cases of hypopigmentation with tetraploid–diploid mosaicism have been documented, both of which were described as hypomelanosis of Ito 10, 23. In both cases, the patients had blood karyotypes that were normal with subsequent skin biopsy chromosome analysis demonstrating tetraploid mosaicism. Our patient is a rare survivor of tetraploid mosaicism with scalp hair hypopigmentation and combined dermatologic hypopigmentation and hyperpigmentation. Signs of pigmentary dysplasia, especially the presence of coexistent hypo/hyperpigmentation, should initiate an evaluation with chromosome analysis in skin fibroblasts.

In conclusion, we report on the first case of tetraploid/diploid mosaicism with pigmentary changes involving both hair and skin. Our report supplements the literature with a new phenotypic expression of tetraploid–diploid mosaicism. Our report also characterizes the wide variety of pathology affecting multiple organ systems in this patient, demonstrating the necessity of intensive multidisciplinary and subspecialty support to optimize care for patients with tetraploid–diploid mosaicism.

## Conflict of Interest

The authors have no conflict of interest, and there was no financial support. The views expressed in this article are those of the author and do not necessarily reflect the policy or position of the Department of the Army, Air Force, Department of Defense, nor the US government. We certify that all individuals who qualify as authors have been listed; each has participated in the conception and design of this work, the analysis of data (when applicable), the writing of the document, and the approval of the submission of this version; that the document represents valid work; that if we used information derived from another source, we obtained all necessary approvals to use it and made appropriate acknowledgements in the document; and that each takes public responsibility for it.

## Authorship

JPS: expanded the draft version from three pages to its current form by performing an extensive chart review on the patient and revised literature review, made corrections per editing authors, and submitted the article for publication. EF: wrote an initial 3‐page draft for the article and performed initial literature review. JSH: cared for the patient as a geneticist, reviewed the article, and recommended edits which were implemented. LOR: cared for the patient as a geneticist, reviewed the article, and recommended edits which were implemented and provided guidance on submission process for publication.

## References

[1] Ozler, S. , A. O. Ersoy , E. Oztas , V. Topcu , S. Celen , and N. Danisman . 2015. The unprecedented recurrent diploid/tetraploid mosaicism of trisomy‐18 (mixoploidy; 4n+18/2n+18): clinical report. Am. J. Med. Genet. Part A 167A:1650–1653. 10.1002/ajmg.a.37062 25851783

[2] Stefanova, I. , J. Jenderny , E. Kaminsky , A. Mannhardt , P. Meinecke , L. Grozdanova , et al. 2010. Mosaic and complete tetraploidy in live‐born infants: two new patients and review of the literature. Clin. Dysmorphol. 19:123–127. 20305547 10.1097/MCD.0b013e3283353877

[3] Edwards, M. , J. P. Park , D. H. Wurster‐Hill , and J. M. Graham . 1994. Mixoploidy in humans: two surviving cases of diploid‐tetraploid mixoploidy and comparison with diploid‐triploid mixoploidy. Am. J. Med. Genet. 52:324–330. 7810564 10.1002/ajmg.1320520314

[4] Alonso, L. , I. Melaragno , A. Borolai , S. Takeno , and D. Brunoni . 2002. Tetraploid/diploid mosaicism: case report and review of the literature. Ann. Genet. 45:177–180. 12668163 10.1016/s0003-3995(02)01137-1

[5] Aughton, D. J. , H. M. Saal , J. A. Delach , Z. Rahman , and D. Fisher . 1988. Diploid/tetraploid mosaicism in a liveborn Infant demonstrable only in the bone marrow: case report and literature review. Clin. Genet. 33:299–307. 3282728 10.1111/j.1399-0004.1988.tb03452.x

[6] Cavalanti, D. P. , and L. M. Zanchetta . 2005. Interphase‐FISH study in three patients with tetraploid/diploid Mosaicism. Eur. J. Med. Genet. 48:41–50. 15953405 10.1016/j.ejmg.2005.01.009

[7] Hochberg, J. C. , P. M. Miron , B. N. Hay , B. A. Woda , S. A. Wang , M. Richert‐Przygonska , et al. 2008. Mosaic tetraploidy and transient GFI1 mutation in a patient with severe chronic neutropenia. Pediatr. Blood Cancer 50:630–632. 17096407 10.1002/pbc.21094

[8] Kelly, T. E. , and J. M. Rary . 1974. Mosaic tetraploidy in a two‐year‐old female. Clin. Genet. 6:221–224. 4426137 10.1111/j.1399-0004.1974.tb00655.x

[9] Kohn, G. , B. H. Mayall , M. E. Miller , and W. J. Mellman . 1967. Tetraploid‐diploid mosaicism in a surviving infant. Pediatr. Res. 1:461–469. 4171101 10.1203/00006450-196711000-00005

[10] Leonard, N. J. , and D. J. Tomkins . 2002. Diploid/tetraploid/t(1;6) mosaicism in a 17‐year‐old female with hypomelanosis of Ito, multiple congenital anomalies, and body asymmetry. Am. J. Med. Genet. 112:86–90. 12239727 10.1002/ajmg.10662

[11] Olgun‐Erdemir, E. , M. S. Yildirim , and M. Karsiyaka . 2010. Generalized aggressive periodontitis in a child with 92, XXYY/46, XY mosaicism: report of a second case. Turk. J. Pediatr. 52:94–96. 20402075

[12] Quiroz, E. , A. Orozco , and F. Salamanca . 1985. Diploid‐tetraploid mosaicism in a malformed boy. Clin. Genet. 27:183–186. 3978853 10.1111/j.1399-0004.1985.tb00208.x

[13] Reddy, C. M. , D. N. Singh , and P. Crump . 1977. Diploid‐tetraploid mosaicism in an offspring of a 46, XX/47, XXX mosaic mother. J. Natl Med. Assoc. 69:563–564. 904007 PMC2609611

[14] Rojanasakul, A. K. , H. Gustavson , H. Lithell , and S. J. Nilius . 1985. Tetraploidy in two sisters with the polycystic ovary Syndrome. Clin. Genet. 27:167–174. 3978852 10.1111/j.1399-0004.1985.tb00206.x

[15] Scarbrough, P. R. , J. Hersh , M. K. Kukolich , A. J. Carrol , S. C. Finely , R. Hochberger , et al. 1984. Tetraploidy: a report of three live‐born infants. Am. J. Med. Genet. 19:29–37. 6496571 10.1002/ajmg.1320190106

[16] Sharma, A. , P. Paliwal , V. Dadhwal , Y. Sharma , and D. Deka . 2009. Rare finding of 2n/4n mixoploidy in mother and fetus with severe immune hydrops. Cytogenet. Genome. Res. 124:90–93. 19372673 10.1159/000200092

[17] Sousa, M. G. F. , L. M. A. Moreira , L. M. Freitas , L. I. S. Peixoto , and V. R. Silva . 1996. Report of a diploid: tetraploid liveborn Infant. Braz. J. Genet. 19:365–369.

[18] Tozum, T. F. , E. Berker , H. Akincibay , O. Ozer , D. Aktas , I. Tezcan , et al. 2005. Tetraploid/diploid mosaicism with generalized aggressive periodontitis. J. Periodontal. 76:1567–1571. 10.1902/jop.2005.76.9.1567 16171449

[19] Veenema, H. , E. W. Tasseron , and J. P. Geraedts . 1982. Mosaic tetraploidy in a male neonate. Clin. Genet. 22:295–298. 7160100 10.1111/j.1399-0004.1982.tb01842.x

[20] Wilson, G. N. , M. J. Vekemans , and P. Kaplan . 1988. MCA/MR syndrome in a female infant with tetraploidy mosaicism: review of the human polyploidy phenotype. Am. J. Med. Genet. 30:953–961. 3055989 10.1002/ajmg.1320300413

[21] Wittwer, B. B. , and H. B. Wittwer . 1989. Dermatoglyphic features of a male with diploid/tetraploid mosaicism. Clin. Genet. 35:310–311. 2714017 10.1111/j.1399-0004.1989.tb02949.x

[22] Wullich, B. , W. Henn , E. Groterath , A. Ermis , S. Fuchs , and M. Zanki . 1991. Mosaic tetraploidy in a liveborn infant with features of the DiGeorge anomaly. Clin. Genet. 40:353–357. 1756611 10.1111/j.1399-0004.1991.tb03109.x

[23] Vormittag, W. , C. Ensinger , and M. Raff . 1992. Cytogenetic and dermatoglyphic findings in a familial case of Hypomelanosis of Ito (incontinentia pigmenti achromians). Clin. Genet. 41:309–314. 1623628 10.1111/j.1399-0004.1992.tb03404.x

[24] Nehal, K. , R. PeBenito , and S. Orlow . 1996. Analysis of 54 cases of hypopigmentation and hyperpigmentation along the Lines of Blaschko. Arch. Dermatol. 132:1167–1170. 8859026

